# Audit of Admissions to Acute Psychiatric Inpatient Unit of Patients With Eating Disorders/Disordered Eating

**DOI:** 10.1192/bjo.2024.626

**Published:** 2024-08-01

**Authors:** Roopa Sanglikar, Gabriela Martyn

**Affiliations:** Norfolk and Suffolk NHS Foundation Trust, Bury St Edmunds, United Kingdom

## Abstract

**Aims:**

The aim of this study was to understand current clinical practice, adherence to evidence-based guidelines, and the perceptions, knowledge and attitudes of the multidisciplinary team caring for inpatients with eating disorder/disordered eating on general adult psychiatric ward.

**Methods:**

The audit was undertaken at inpatient general adult psychiatry ward between 1st July 2022 to 30th April 2023. A retrospective method was used to collect data on admissions of patients with eating disorder/disordered eating alongside qualitative data retrieved for perceptions, knowledge and attitudes of the multidisciplinary team (MDT) and use of and adherence to national guidelines. The data was collected from everyday bed state and MDT handover, admission summary, electronic notes which included physical health charts and discharge summaries. The MDT staff involved were nurses, doctors, health care assistants, dieticians, psychologist, and occupational therapist.

Patients were included if eating disorder management was indicated and undertaken at some stage during the admission, even if the eating disorder was not the primary reason for admission. The age group was above 18 years and included male and female patients. Eight discrete admissions (6 females, 1 male and 1 transgender patient) were included in the audit. Adverse events like refeeding syndrome, electrolyte derangement needing Intravenous/Nasogastric tube feeding, self-harm, level of cooperation between medical and community eating disorders team, community mental health teams and outcomes were recorded. Data analysis was done through Microsoft Excel. Percentages of patients who had met each of the standards were calculated. Documented practices were compared in line with standards of NICE (National Institute for Health and Care Excellence) guidelines and MEED (Medical Emergencies in Eating Disorders) guidelines.

**Results:**

The audit concluded that gaps exist between evidence-based practice and patient care. Despite being admitted due to concern about eating difficulties, a substantial number of patients were not given an eating disorder diagnosis on discharge. And the patients who had eating disorder as primary diagnosis had limited inreach support from specialist team.

**Conclusion:**

There is major challenge in management of disordered eating presentations within inpatient general adult psychiatry units and inreach specialist support for those admitted with eating disorders as primary diagnosis. These findings emphasize for targeted implementation strategies to improve patient care and uptake of research into practice.

